# The Potential Role of Placenta Morphological, Cytogenetic and Histological Findings in Fetal Phenotype Modeling: A Case Report with Unanswered Questions

**DOI:** 10.15388/Amed.2025.32.1.8

**Published:** 2025-02-18

**Authors:** Estere Strautmane, Liene Korņejeva, Dzintra Ločmele, Marta Piksena, Diāna Bokučava, Natālija Vedmedovska

**Affiliations:** 1 Rīga Stradiņš University, Faculty of Residency in Obstetrics and Gynecology, Riga, Latvia; 2 Riga Maternity Hospital, Riga, Latvia; 3 Clinic of Medical Genetics and Prenatal Diagnostics, Children’s Clinical University Hospital, Riga, Latvia; 4 Children’s Clinical University Hospital , Department of Pathology, Riga, Latvia

**Keywords:** fetal growth restriction, small-for-gestational-age, chorionic villus sampling, intraplacental hemorrhage, intervillous thrombi, perivillous fibrin deposition, vaisiaus augimo atsilikimas, mažas gestacinis amžius, choriono gaurelių mėginių ėmimas, intraplacentinis kraujavimas, tarpvietės trombai, perivilus fibrinogeno nusėdimas

## Abstract

Placental abnormalities significantly contribute to fetal mortality. Maternal vascular underperfusion emerges as the prevailing diagnosis. At the same time, genomic alterations within the placenta might play a role in the development of placental dysfunction. We present a case report of a 24-year-old primigravida with an uneventful medical history, admitted at 21 weeks and 2 days gestation. Despite a low risk for typical trisomies, a high risk for preeclampsia and fetal growth restriction (FGR) was recognized in the first trimester. Anhydramnios, abnormal placental morphology, retrochorial and retromembranous hematomas were observed, prompting the termination of pregnancy (TOP). Histological examination revealed multiple placental abnormalities, while the fetus displayed normal anatomy and phenotype. Discussion encompasses the varied manifestations of histopathological findings, and potential associations with adverse pregnancy outcomes. Our case underscores the importance of meticulous evaluation and multidisciplinary collaboration in managing pregnancies with placental anomalies. Further research is crucial to discern the intricate relationship between placental cytogenetics, morphology, and pregnancy outcomes, thus facilitating better clinical management and counseling strategies.

## Abstract (it)

Placentos anomalijos labai prisideda prie vaisiaus mirtingumo. Vyraujanti diagnozė – motinos kraujagyslių nepakankama perfuzija. Tuo pat metu placentoje esantys genomo pokyčiai gali turėti įtakos placentos disfunkcijos vystymuisi. Pateikiame 24 metų pirmakartės gimdyvės, kurios ligos istorija nesudėtinga ir kuri buvo paguldyta į ligoninę 21 nėštumo savaitės ir 2 dienų, atvejo aprašymą. Nepaisant mažos dažniausių trisomijų rizikos, pirmajame nėštumo trimestre buvo nustatyta didelė preeklampsijos ir vaisiaus augimo atsilikimo (FGR) rizika. Nustatytas anhidramnionas, pakitusi placentos morfologija, retrochorialinės ir retromembraninės hematomos, todėl buvo nuspręsta nutraukti nėštumą (TOP). Histologinis tyrimas atskleidė daugybę placentos anomalijų, o vaisiaus anatomija ir fenotipas buvo normalūs. Diskusijoje aptariami įvairūs histopatologinių tyrimų rezultatai ir galimos sąsajos su neigiamais nėštumo rezultatais. Mūsų atvejis pabrėžia kruopštaus įvertinimo ir daugiadalykinio bendradarbiavimo svarbą gydant nėščiąsias su placentos anomalijomis. Būtina tęsti mokslinius tyrimus, kad būtų galima išsiaiškinti sudėtingą placentos citogenetikos, morfologijos ir nėštumo baigties ryšį, taip palengvinant geresnį klinikinį valdymą ir konsultavimo strategijas.

## 1. Introduction

The placenta serves as a vital lifeline, facilitating the nourishment and substance necessary for the optimal fetal growth and development during the course of the pregnancy. Placental abnormalities significantly contribute to fetal mortality, with lesions suggestive of maternal vascular insufficiency playing a critical role in this outcome. Therefore, examination of the placenta can unveil a myriad of pathological conditions, extending from subtle aberrations to significant anomalies, offering crucial insights into the health and development of both the fetus and the mother. In pregnancies at gestational ages up to 24 weeks, undetermined causes of a fetal negative outcome stand out as the primary concern, with placental lesions emerging as a consequential secondary factor in the understanding of perinatal mortality [1]. Maternal vascular underperfusion emerges as the prevailing diagnosis drawn by pathologists with a notable frequency, but, unfortunately, none of the findings show superior reliability and reproducibility [2]. Cytogenetic examination of the placenta is conducted even more infrequently, representing an underutilized diagnostic path which is denoted by a significant potential in unraveling the genetic ground of various placental abnormalities and shedding light on their implications for the pregnancy outcome. At the same time, genomic alterations within the placenta might play a role in the development of placental dysfunction. It is known that *confined placental mosaicism* (CPM) occurs when the placental tissue has an abnormal number of chromosomes with the presence of karyotypically normal fetus, which is verified by comparing chorionic villi and *amniotic fluid* (AF) [3-6]. Mosaicism can be a result of either a faulty mitotic cell division or a meiotic error culminating in trisomy with the subsequent loss of the supernumerary chromosome, which occurs during the embryonic, fetal development, or even later. It refers to the presence of two or more cell lines in the tissue which differ in genotype but are derived from the same zygote [5-7]. In most cases, CPM does not cause any pregnancy-related complications. However, certain authors have reported a significant correlation between CPM and unfavorable pregnancy and postnatal outcomes, such as *fetal growth restriction* (FGR), the birth of *small-for-gestational-age* (SGA) neonates, fetal loss, and preterm delivery [4,7], while others have opposed this association [6,8]. This discrepancy may be attributable to different aneuploidies causing each specific level of placental function alteration. Furthermore, histopathological examination of mosaic placenta does not reveal any specific abnormalities [9,10].

Meanwhile, numerous common placental disorders remain insufficiently studied, and are sometimes controversial among ultrasound practitioners. For instance, differentiating between placental hematoma, massive perivillous fibrin deposition, and placental infarction is challenging based solely on ultrasound imaging [11]. These conditions may present as hyperechoic or echolucent regions within the placenta that are devoid of blood flow. All the three issues are linked to negative pregnancy outcomes. However, massive perivillous fibrin deposition is associated not only with increased risks of fetal growth restriction, fetal death, and habitual miscarriages, but also with recurrent risk in subsequent pregnancies in one third of the cases [12].

Cystic lesions of the placenta, which can appear as echolucent areas with or without an echogenic border in the absence of blood flow, may pathologically correspond to decidual septal cysts, intervillous thrombi, or infarctions, potentially leading to complications related to placental insufficiency [13]. It is of importance to differentiate cystic lesions from placental mesenchymal dysplasia, which is frequently misdiagnosed as a partial mole, placental mosaicism, subchorionic hematoma, placental infarcts, as well as spontaneous abortion with hydropic changes [13].

We demonstrate a case of a morphologically and histologically abnormal placenta with a structurally normal fetus. Different ultrasound features of variety abnormalities combined in one single case and constituted the diagnostic dilemma for professionals, resulting in a prognostic challenge. A detailed description of lesions can improve the understanding of their causes and their impact on the rate of particular adverse pregnancy outcomes.

## 2. Case Presentation

A 24-year-old healthy primigravida at 21 weeks and 2 days gestation with an uneventful medical and family history was admitted at Riga Maternity Hospital due to complaints of a watery-bloody vaginal discharge for a month, specifically, since the 15^th^ week of pregnancy.

The first trimester screening demonstrated a low risk for typical trisomies. All chromosomal markers on expert ultrasound were negative. The PAPP-A level was 0.75 MOM and HCG 1.6 MOM, the *nuchal translucency* (NT) was -1.8 mm, the nasal bone was reported as present, the ductus venosus a-wave and the blood flow through the tricuspid valve illustrated a normal pattern. A scrutinized fetal anatomic survey did not reveal any structural anomalies [14]. The uterine artery *pulsatility index* (PI) was bilaterally increased, and the reported mean PI constituted 1.48 MOM. Hence, the predicted risk for common trisomies was low, but the risk for preeclampsia and FGR was estimated as high [15], and 150 mg of aspirin was administered [16].

Following the 1^st^ trimester ultrasound, the patient was examined three times in another institution due to persistent spotting. During the examination, the placenta was described as hypertrophic, and persisted retroamnial hematoma was documented. Secondary anemia was revealed. Aspirin therapy was halted at 17+3 weeks of gestation.

At 20 weeks and 4 days, a fetal anomaly scan was performed at Riga Maternity Hospital by a maternal-fetal medicine specialist [17,18]. The fetus corresponded to 19 weeks and 1 day of gestation. The evaluation was restricted due to anhydramnios, but fetal structures and genetic markers were absent, and the urinary bladder and the heart appeared normal on ultrasound.

**Figure 1:**
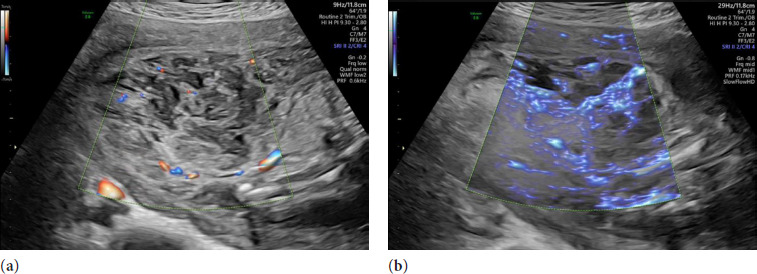
Ultrasound findings of placenta: (**a**) Placental lacunae consisting of an irregular area of low echogenicity in the placental parenchyma; (**b**) *Superb microvascular imaging* (SMI) of the overperfused placental area

The placental plate was located at the fundus and laterally on the right side of the uterus. Morphologically, it looked severely abnormal: retrochorial and retromembranous hematoma along the entire anterior wall measuring 5.9 x 7.5 x 5.9 cm (thus spatially constituting 139 cm^3^) was visualized, and overperfused area, huge lakes, and a ‘net-like’ zone with vascularisation in structures that resembled ‘septae’ (see Figure 1) was seen. Multiple cystic areas were also visualized on ultrasound examination in some parts of the placenta.

**Figure 2:**
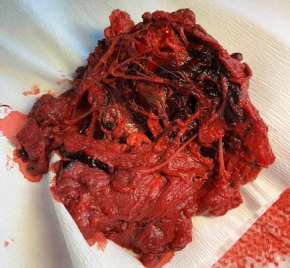
Macroscopic appearance of the placenta with massive intravillous thrombi and intraplacental hemorrhage

During the examination per speculum, watery-bloody vaginal discharge was observed, an *Actim PROM* test was performed, and it appeared to be positive, thus confirming the diagnosis of a *preterm premature rupture of membranes* (PPROM). Taking into account the gestational age of 21 weeks and 2 days, ruptured membranes and anhydramnios prognosis were considered as serious. The parents opted for the *termination of pregnancy* (TOP). For that reason, the patient was hospitalized, and TOP was induced with sublingual *Misoprostol* (400 µg every 3 hours from 3 pm). A complete blood count with leukocyte formula, coagulation tests and C-reactive protein (CRP) were obtained. The results were as follows: red blood cells – 3.56 x 10^12/L, hemoglobin – 11.7 g/dL, hematocrit – 35.3%, platelets – 538 x 10^9/L, leukocytes – 13.59 x 10^9/L with neutrophilia – 10.26 10^9/L, *Activated Partial Thromboplastin Time* (APTT) – 22.4 s, fibrinogen – 6.6 g/L and CRP-3.57 mg/L. The patient reported severe pain at 6pm and requested epidural anesthesia. In 45 minutes, a male fetus was spontaneously born weighing 258 grams. Dilation and curettage of the uterine cavity were performed. Macroscopically fetal membranes and placental tissues demonstrated signs of necrosis, a massive hemorrhage (the weight of thrombi up to 48g). The weight of placenta was 185g (see Figure 2). The aborted fetus and placental tissues were sent to Children’s Clinical University Hospital, Riga, Latvia for histological examination. A sample of chorion was sent for cytological examination.

The blood loss during the procedure was 200 ml. The patient received 10 IU Oxytocin intravenously for the prevention of hemorrhage. Intravenous antibacterial treatment with Amoxicillin-Clavulanic acid 1.2 g and Clindamycin 900 mg was continued for 7 days after the abortion up until vaginal discharges had become minimal and light. A day after TOP, red blood cells revealed – 3.08 x 10^12/L, hemoglobin – 10.2 g/dL, hematocrit – 31.0%, platelets – 432 x 10^9/L, leukocytes – 8.83 x 10^9/L, and CRP – 24.07 mg/L. Antianemic treatment was continued, and, after 3 days, red blood cells unveiled 3.12 x 10^12/L, hemoglobin – 10.3 g/dL, hematocrit – 31.3%, platelets – 409 x 10^9/L, leukocytes – 6.47 x 10^9/L, CRP – 9.91 mg/L. In one week after TOP, the ultrasound image corresponded to medical condition, and the patient was discharged in a satisfactory condition.

**Figure 3:**
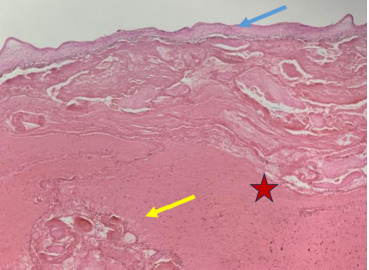
Histological cross section of placenta. Blue arrow – chorionic plate, red star – massive subhorionic and intraplacental hemorrhage, yellow arrow – entrapped ischemic villi

**Figure 4:**
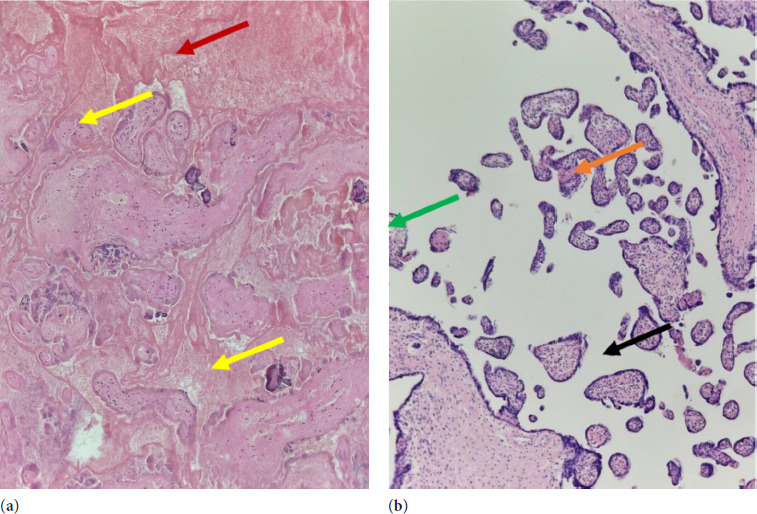
Histological cross-section of chorionic villi: (**a**) Entrapped necrotic (ischemic), avascular villi (yellow arrow), surrounded by chronic, organized hematoma (red arrow); (**b**) Hypermatured terminal villi (green arrow) with intravillous (orange arrow) and extravillous (black arrow) fibrin deposition

**Figure 5:**
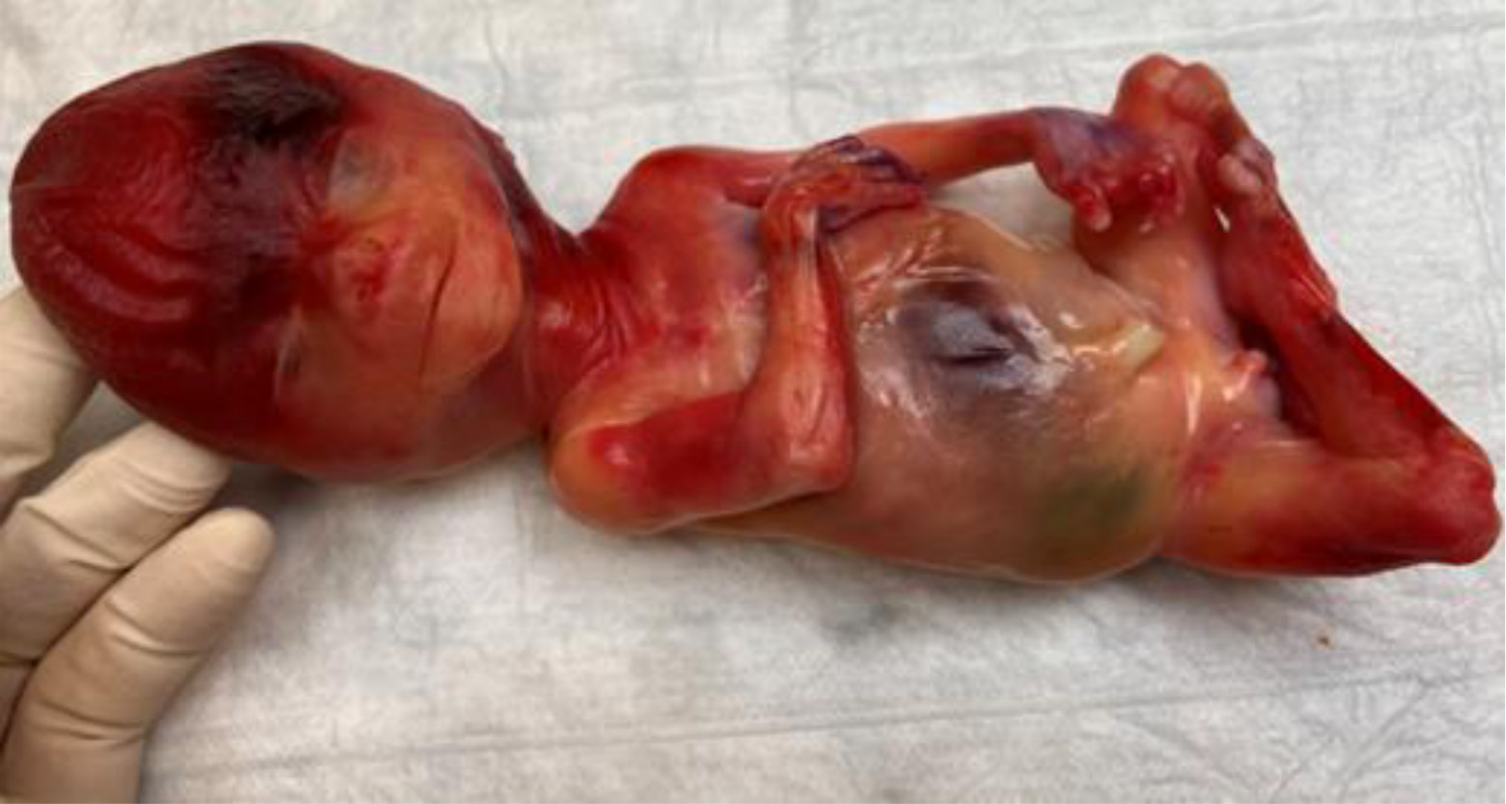
A male fetus without prominent dysmorphic features, with measurements 24 cm, 258 g (normal in 21 gestation weeks: 387+/-107g), indicating intrauterine growth restriction

The histological evaluation of the placenta revealed intraplacental thrombosis and hemorrhage around villi, massive fibrine deposition, as well as villous fibrosis (see Figure 3 and Figure 4). The growth-restricted male fetus demonstrated a normal anatomy and phenotypical features (see Figure 5).

The cytogenetic examination of chorionic villi was performed by using Cytocell Fast FISH and the GTG (G-bands by trypsin using Giemsa) staining method. The FISH result demonstrated normal XY sex chromosomes and excluded trisomies 13, 18 and 21. In culture 30 metaphases, (300–400 G-bands) were analyzed by the standard method. 3 metaphases showed 45,X karyotype; a reciprocal balanced translocation with the normal male sex karyotype was found in 5, and the remaining 22 carried a normal male karyotype with no additional aberrations: 45,X[3]/46,XY,t(2;14)(q31;q32)[5]/46,XY[22]. The cells were grown on 2 culture media; all aberrant metaphases came from one culture.

The investigation of parental karyotypes revealed normal results, excluding maternal interfering.

## 3. Discussion

Maternal vascular pathology and other alterations linked to superficial placental implantation are hypothesized to serve as underlying risk factors for the unfavorable pregnancy outcome, while modifications in the overlying villous parenchyma and the intervillous space are not regarded as contributing factors to the condition [2]. In terms of the villous and intervillous lesion that is described in cases of maternal underperfusion, intervillous fibrin showed the strongest correlation with chronic subgroups of severe underperfusion [19], but this criterion often shows some diagnostic uncertainty and poor reproducibility [2]. The incidence of MPFD among pregnant women varied from 0.09% to 11.8%, and, according to the definition, 25% of the intervillous spaces should be occupied by fibrin [20,21]. Ultrasound findings suggestive of MPFD include cystic hypoechoic areas surrounded by echogenic tissue with very low or no blood flow in enlarged placenta. Ultimately, the conclusive diagnosis of MPFD can be made solely by the pathological examination. In our specific placental specimen, an increased density of the placental villous stroma was observed, and it may be responsible for the illustrated ultrasound appearance, where cystic regions are surrounded by echogenic areas with minimal blood flow, representing villi compressed by laminated fibrin and erythrocytes [22]. Simultaneously, a sub-chorionic hematoma that can be referred to subchorionic intervillous thrombus [19] is reported in the histology of our case. On ultrasound imaging, their appearance varies depending on the phase of the process from hyperechoic to hypoechoic foci. The most crucial point, acknowledged by all experts, is that placental hematomas never show a signal on color Doppler evaluation [23]. Innovative Doppler techniques, such as *SlowflowHD*®, which we applied in our clinical case, could be helpful to verify the absence of the blood flow around the hypoechoic region [24]. During the present ultrasound examination, there was a slow flow in structures that resembled ‘septas’ in between cystic areas, which misled the diagnosis, and, in some parts, the examination yielded an appearance similar to placental mesenchymal dysplasia. Contrary to other malformations, placental mesenchymal dysplasia is an extremely rare placental abnormality, constituting a form of a congenital mesodermal anomaly. While Doppler techniques are powerful for assessing vascular and hemodynamic conditions in cases of placental infarction or MPFD, their diagnostic utility is limited in mesenchymal dysplasia. In spite of scrupilous histological examination of the placenta in this specific case, pathologists did not reveal any typical features of mesenchymal dysplasia.

Poor remodeling of the uteroplacental spiral arteries is linked to placental abruption, as well as a spontaneous preterm premature rupture of membranes [25]. Contrary to Miura and Wilkins-Haug, we did not detect any abnormal uterine artery blood flow in the second trimester, despite observing a completely pathological placental morphology. This discrepancy suggests an alternative mechanism of placental alteration, potentially involving structural developmental defects rather than the abnormal spiral artery invasion alone. Consequently, the extensive involvement of the placental tissue observed in our case may be partially attributed to placental chromosomal abnormality in its part. The existing literature on CPM demonstrates that mosaicism in the placental tissue can vary in the affected cell quantity, the structural level, the type of aneuploidy, and a potential functional deficit, determining the fate of the developing pregnancy. Although the cytogenetical findings in our case are certainly not the only pathogenetic factor in play, two different cell line formations raise questions about the course of the pathological events.

It should also be noted that aberrant findings in one culture only cannot exclude pseudomosaicism, and therefore, the results should be interpreted cautiously, especially knowing that the FISH test, which was performed for the most common aneuploidies (50 cells tested), failed to show mosaic X chromosome monosomy. Information about the fetal cell karyotype could be extremely helpful in such cases, and a confirmatory invasive diagnostic procedure should be conducted to ascertain the presence or absence of the anomaly before making any decisions regarding the pregnancy. Nevertheless, amniocentesis is regarded as the gold standard for confirming a fetal anomaly. Unfortunately, confirmatory amniocentesis in our case was not possible due to oanhydramnios, and this is the primary limitation of the presently discussed clinical situation. Yet, certainly, our case raises reasonable inquiries regarding the imperative need for a comprehensive cytogenetic examination across various parts of the placenta, particularly in cases exhibiting severe placental abnormalities alongside with aborted fetus tissues storage and investigation. From prior research, it is evident that genomic alterations are not consistently spread throughout placentas, potentially elucidating the diverse pregnancy outcomes observed in cases of a placental chromosomal abnormality.

New technologies, such as cell-based non-invasive prenatal testing, offer the potential to detect CPM and non-invasively pinpoint the specific location of mosaicism. This advancement enhances our understanding of the relationship between genetic changes, placental function, and obstetric outcomes [26].

Finally, *antiphospholipid syndrome* (APS) may be on the list of differential diagnosis in our clinical case, as anticardiolipin IgG and IgM was found to be increased in the serum on the first occasion (36.7.2; normal range ≥40IU/ml) [27], which normalized after 6 month till 23.2 IU/ml; albeit IgG/IgM antiphospholipid antibodies (respectively, IgG 2.8 and IgM 5.8; normal range <10MPLU/ml) and lupus anticoagulant (1.1) were found to be negative. The primary vascular manifestation in APS is thrombus formation, commonly observed in histopathological examinations of placental infarctions. Infarctions in APS cases typically occur later in pregnancy. Moreover, APS is associated with placental hypoperfusion and ischemic changes, contributing to its pathogenicity. However, an increased number of syncytial knots and fibrin deposition, while present, are not specific indicators of APS [28]. As intervillous hematomas and villous infarction may correlate with maternal thrombophilia, screening for a genetically predisposed clotting tendency is highly advisable. However, in the current clinical scenario, such screening yielded negative results. Additionally, despite the early initiation of a low-dose aspirin therapy in the first trimester to mitigate the pregnancy complications, the patient did not respond to prophylaxis favorably.

In spite of the existing data, we can neither define the risk for recurrency in a case of placental infarction and/or hematomas, nor can we apply any validated methods of the prevention of massive deposition of fibrin. Of course, histologic lesions should not be assessed in isolation, but rather within the broader context of clinical findings and a maternal-fetal health indicator.

Our case endorses the idea that certain extensive histological and genetic tests for the placenta may be more cost-effective than others, and might be chosen based on specific situations. Comprehensive studies on health economics are necessary to identify the most suitable investigations.

## 4. Conclusions

Overall, this case report highlights the importance of a comprehensive approach involving cytogenetic, molecular, and pathological investigations to guide clinical decision-making and provide personalized counseling for pregnancies affected by placental morphological anomalies and/or mosaic, or full aneuploidy. Further research is warranted to elucidate the optimal management strategies for cases with gross structural placental defects, consultation about a reduced recurrency risk with the aim to improve the pregnancy outcomes and precipitate possible complications, associated with the gestational period.

## References

[1] Roescher AM, Timmer A, Erwich JJ, Bos AF (2014). Placental pathology, perinatal death, neonatal outcome, and neurological development: a systematic review. PLoS One.

[2] Redline RW, Boyd T, Campbell V (2004). Maternal vascular underperfusion: nosology and reproducibility of placental reaction patterns. Pediatr Dev Pathol.

[3] Kalousek DK, Vekemans M (1996). Confined placental mosaicism. J Med Genet.

[4] Toutain J, Goutte-Gattat D, Horovitz J, Saura R (2018). Confined placental mosaicism revisited: Impact on pregnancy characteristics and outcome. PLoS One.

[5] Spinillo SL, Farina A, Sotiriadis A (2022). Pregnancy outcome of confined placental mosaicism: meta-analysis of cohort studies. Am J Obstet Gynecol.

[6] Grati FR, Ferreira J, Benn P (2020). Outcomes in pregnancies with a confined placental mosaicism and implications for prenatal screening using cell-free DNA. Genet Med.

[7] Eggenhuizen GM, Go A, Koster MPH, Baart EB, Galjaard RJ (2021). Confined placental mosaicism and the association with pregnancy outcome and fetal growth: a review of the literature. Hum Reprod Update.

[8] Baffero GM, Somigliana E, Crovetto F (2012). Confined placental mosaicism at chorionic villous sampling: risk factors and pregnancy outcome. Prenat Diagn.

[9] Guenot C, Kingdom J, De Rham M (2019). Placental mesenchymal dysplasia: An underdiagnosed placental pathology with various clinical outcomes. Eur J Obstet Gynecol Reprod Biol.

[10] Maywald R, Bryan J, Peters M, Armes J, Lourie R (2012). Histopathological or clinical features do not predict the presence of high level tetraploidy as a form of confined placental mosaicism in pregnancies affected by otherwise idiopathic IUGR. Pathology.

[11] Delcominette S, Chantraine F (2022). Placental mesenchymal dysplasia. *Visual Encyclopedia of Ultrasound in Obstetrics and Gynecology*.

[12] Andres RL, Kuyper W, Resnik R, Piacquadio KM, Benirschke K (1990). The association of maternal floor infarction of the placenta with adverse perinatal outcome. Am J Obstet Gynecol.

[13] Harris RD, Cho C, Wells WA (1996). Sonography of the placenta with emphasis on pathological correlation. Semin Ultrasound CT MR.

[14] Bilardo CM, Chaoui R, International Society of Ultrasound in Obstetrics and Gynecology (2023). ISUOG Practice Guidelines (updated): performance of 11-14-week ultrasound scan. Ultrasound Obstet Gynecol.

[15] The Fetal Medicine Foundation Assessment of risk for preeclampsia (PE). https://fetalmedicine.org/research/assess/preeclampsia/background.

[16] The Fetal Medicine Foundation Tricuspid flow. https://www.fetalmedicine.org/fmf-certification-2/tricuspid-flow.

[17] Salomon LJ, Alfirevic Z, Berghella V (2022). ISUOG Practice Guidelines (updated): performance of the routine mid-trimester fetal ultrasound scan. Ultrasound Obstet Gynecol.

[18] Carvalho JS, Allan LD, International Society of Ultrasound in Obstetrics and Gynecology (2013). ISUOG Practice Guidelines (updated): sonographic screening examination of the fetal heart. Ultrasound Obstet Gynecol.

[19] Altshuler G (1993). A conceptual approach to placental pathology and pregnancy outcome. Semin Diagn Pathol.

[20] Kim EN, Lee JY, Shim JY (2019). Clinicopathological characteristics of miscarriages featuring placental massive perivillous fibrin deposition. Placenta.

[21] Spinillo A, Gardella B, Muscettola G, Cesari S, Fiandrino G, Tzialla C (2019). The impact of placental massive perivillous fibrin deposition on neonatal outcome in pregnancies complicated by fetal growth restriction. Placenta.

[22] Hernandez-Andrade E, Huntley ES, Bartal MF (2022). Doppler evaluation of normal and abnormal placenta. Ultrasound Obstet Gynecol.

[23] Neville G, Russell N, O’Donoghue K, Fitzgerald B (2019). Rounded intraplacental hematoma-A high risk placental lesion as illustrated by a prospective study of 26 consecutive cases. Placenta.

[24] Hasegawa J, Suzuki N (2016). SMI for imaging of placental infarction. Placenta.

[25] Staff AC, Fjeldstad HE, Fosheim IK (2022). Failure of physiological transformation and spiral artery atherosis: their roles in preeclampsia. Am J Obstet Gynecol.

[26] Vestergaard EM, Singh R, Schelde P (2017). On the road to replacing invasive testing with cell-based NIPT: Five clinical cases with aneuploidies, microduplication, unbalanced structural rearrangement, or mosaicism. Prenat Diagn.

[27] Limper M, de Leeuw K, Lely AT (2019). Diagnosing and treating antiphospholipid syndrome: a consensus paper. Neth J Med.

[28] Gerardi MC, Fernandes MA, Tincani A, Andreoli L (2018). Obstetric Anti-phospholipid Syndrome: State of the Art. Curr Rheumatol Rep.

